# Neurotrophin and Adipokine Signatures Associated with Visceral Adiposity-Driven Cardiometabolic and Endocrine Risk in Polycystic Ovary Syndrome

**DOI:** 10.3390/ijms27052440

**Published:** 2026-03-06

**Authors:** Daniela Koleva-Tyutyundzhieva, Maria Ilieva-Gerova, Elena Becheva, Tanya Deneva, Maria Orbetzova

**Affiliations:** 1 Department of Endocrinology and Metabolic Diseases, Faculty of Medicine, Medical University of Plovdiv, 4002 Plovdiv, Bulgaria; mariyail.ilieva@phd.mu-plovdiv.bg (M.I.-G.); elena.becheva@phd.mu-plovdiv.bg (E.B.); maria.orbetzova@mu-plovdiv.bg (M.O.); 2 Department of Clinical Laboratory, Faculty of Medicine, Medical University of Plovdiv, 4002 Plovdiv, Bulgaria; tanya.deneva@mu-plovdiv.bg

**Keywords:** polycystic ovary syndrome, visceral adiposity index, cardiometabolic risk, neurotrophins, BDNF, NGFβ, adipokines, insulin resistance, estradiol

## Abstract

Polycystic ovary syndrome (PCOS) is a heterogeneous endocrine–metabolic disorder associated with insulin resistance (IR), visceral adiposity, and increased cardiometabolic risk. The visceral adiposity index (VAI) is a validated surrogate marker of adipose tissue dysfunction, but its relationship with circulating neurotrophins and adipokine balance in PCOS remains incompletely understood. In this study, 100 women with PCOS were stratified into lower- (n = 50) and higher-risk (n = 50) groups according to VAI. Anthropometric measures, fasting glucose and insulin concentrations, lipid profile, and serum levels of brain-derived neurotrophic factor (BDNF), nerve growth factor-β (NGFβ), leptin, adiponectin, and resistin were assessed. HOMA-IR, adipokine ratios and atherogenic indices were calculated. Multivariate regression showed that BDNF was independently associated with VAI and non-HDL cholesterol, whereas NGFβ was independently linked to HDL cholesterol and estradiol, highlighting neurotrophin relationships with metabolic and endocrine parameters beyond general adiposity. Correlation heatmap and network analyses demonstrated interconnected clusters linking visceral adiposity, IR, dyslipidemia, adipokine imbalance, and neurotrophins, with the leptin/adiponectin ratio emerging as a central integrative marker. These findings suggest that within a PCOS population, VAI-defined cardiometabolic risk is associated with distinct neurotrophin–adipokine signatures, highlighting neurotrophin–adipokine networks underlying visceral adiposity-driven cardiometabolic and endocrine risk.

## 1. Introduction

Polycystic ovary syndrome (PCOS) is one of the most common endocrine disorders in women of reproductive age, affecting approximately 10–15% of the female population worldwide [1,2]. Beyond its reproductive manifestations, PCOS is increasingly recognized as a complex metabolic *condition* characterized by insulin resistance (IR), dyslipidemia, visceral adiposity, and elevated cardiometabolic risk, which substantially contributes to the long-term development of type 2 diabetes mellitus and cardiovascular disease [3,4,5].

Visceral adiposity plays a pivotal role in the metabolic derangements associated with PCOS. Dysfunctional visceral adipose tissue acts as an active endocrine organ, secreting adipokines and bioactive mediators that influence insulin sensitivity, lipid metabolism, and low-grade inflammation [6,7]. The visceral adiposity index (VAI), which integrates anthropometric measures and lipid parameters, has emerged as a practical surrogate marker of visceral fat dysfunction and cardiometabolic risk, potentially outperforming body mass index (BMI) in women with PCOS [8,9].

Visceral adipose tissue is not merely an energy store but a metabolically active endocrine organ, marked by altered adipokine secretion, chronic low-grade inflammation, and impaired insulin signaling [10]. These disturbances create a complex biochemical environment that can affect distant endocrine organs, including the ovary. In women with PCOS, this metabolically adverse milieu likely contributes to systemic IR and ovarian dysfunction [11]. Identifying molecular mediators that link visceral adiposity to metabolic and reproductive abnormalities is therefore crucial to understanding the heterogeneity of cardiometabolic risk in PCOS.

Hyperandrogenism, a hallmark feature of PCOS, further amplifies this interplay through a bidirectional relationship with visceral adiposity. Androgen excess promotes preferential central fat accumulation and worsens IR, while dysfunctional adipose tissue enhances ovarian steroidogenesis and androgen production. This reciprocal interaction establishes a self-perpetuating metabolic–endocrine cycle that reinforces both adipose tissue dysfunction and reproductive disturbances [12].

Within this integrated metabolic–reproductive framework, neurotrophins have emerged as potential molecular intermediates linking metabolic and endocrine regulation. Beyond their classical roles in neuronal growth and survival, neurotrophins are expressed in adipose tissue, the ovary, and peripheral metabolic organs, where they participate in the regulation of energy homeostasis, lipid metabolism, insulin sensitivity, and steroidogenesis. Given this dual metabolic–endocrine functionality, neurotrophins represent biologically plausible candidates that may bridge visceral adipose tissue dysfunction with ovarian alterations in PCOS [13,14].

Among the various members of the neurotrophin family, brain-derived neurotrophic factor (BDNF) and nerve growth factor-β (NGFβ) have received particular attention in metabolic and reproductive research. Experimental evidence, largely derived from animal models and peripheral tissues, suggests that BDNF contributes to energy homeostasis and metabolic regulation; however, direct data on its expression and function in human adipose tissue remain limited [15,16,17]. However, studies investigating circulating BDNF levels in obesity and PCOS have yielded inconsistent results, reporting both increased and decreased concentrations [18,19,20]. In contrast, NGFβ has been more consistently linked to ovarian function and steroidogenesis and may be associated with favorable lipid profiles, yet data regarding its role in cardiometabolic risk in PCOS remain scarce and inconclusive [21,22].

Adipokines such as leptin, adiponectin, and resistin further modulate metabolic and inflammatory pathways in PCOS. Notably, adipokine ratios, including adiponectin/leptin and adiponectin/resistin, have been proposed as sensitive indicators of adipose tissue dysfunction and insulin resistance, providing greater pathophysiological insight than individual adipokine levels alone [23,24,25].

While individual roles of neurotrophins and adipokines in PCOS have been studied, their integrated association with VAI-defined visceral adiposity and cardiometabolic risk remains unexplored in a single cohort. Therefore, the present study aimed to investigate VAI-associated differences in circulating BDNF and NGFβ levels, adipokine ratios, and cardiometabolic parameters in women with PCOS, as well as their relationships with lipid fractions, insulin resistance indices, and estradiol levels. The study was specifically designed to explore metabolic heterogeneity within a well-characterized PCOS cohort stratified by visceral adiposity index, rather than to perform comparisons with non-PCOS controls.

## 2. Results

### 2.1. Clinical and Anthropometric Characteristics According to VAI

No significant differences were observed between the low- and high-VAI PCOS groups with respect to age or height. In contrast, women with high VAI exhibited significantly higher body weight and BMI (*p* < 0.001). Parameters reflecting central adiposity, including waist and hip circumferences, waist-to-hip ratio (WHR), and waist-to-height ratio (WHtR), were all significantly elevated in the high-VAI group (*p* < 0.01–0.001). Additionally, systolic blood pressure (SBP) was significantly higher among women with high VAI (*p* < 0.05), whereas diastolic blood pressure (DBP) did not differ significantly between groups (Table 1).

### 2.2. Adipokines and Neurotrophins

Circulating leptin and resistin concentrations tended to be higher in the high-VAI group (*p* > 0.05), whereas adiponectin levels were significantly lower (*p* = 0.001). Consequently, adiponectin/leptin (A/L) and adiponectin/resistin (A/R) ratios differed markedly between groups, with a shift toward an unfavorable adipokine balance in high-VAI PCOS (*p* < 0.01–0.001). Women with higher VAI exhibited significantly increased circulating BDNF levels, whereas NGFβ concentrations were significantly higher in the low-VAI group (Table 2).

### 2.3. Glucose Homeostasis and Insulin Resistance; Lipid Profile and Atherogenic Indices

Fasting plasma glucose (FBG) levels were comparable between groups. However, women with higher VAI demonstrated significantly elevated fasting immunoreactive insulin (FIRI) concentrations and HOMA-IR values, indicating increased insulin resistance (Table 3). Marked differences in lipid metabolism were observed between VAI groups. The high-VAI group exhibited significantly lower HDL-C and higher TG levels. LDL-C and TC concentrations were comparable; however, non-HDL-C and AIP were significantly elevated in women with higher VAI (Table 3), indicating a more atherogenic lipid phenotype.

### 2.4. Hormonal Parameters

No statistically significant differences in gonadotropin or steroid hormone parameters were observed between PCOS patients with low and high VAI. Estradiol, androgen levels, sex hormone-binding globulin (SHBG), and the free androgen index (FAI) showed only numerical differences without statistical significance (Table 4).

### 2.5. Correlation Analyses

**Figure 1:**
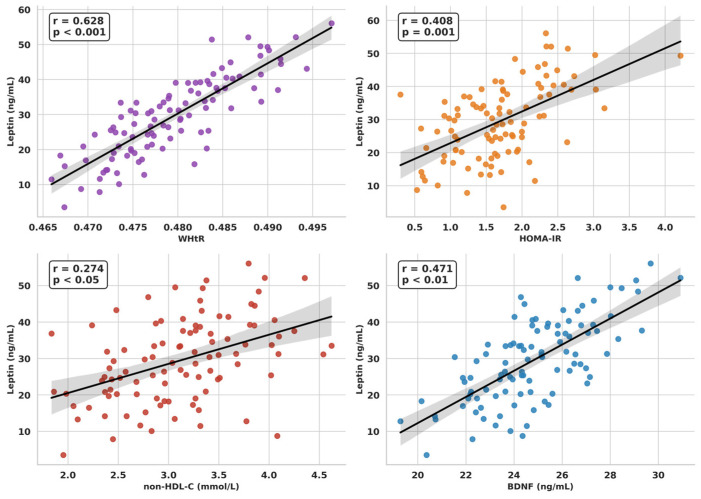
Associations of Leptin with WHtR, HOMA-IR, non-HDL Cholesterol, and BDNF in the Whole Study Group.

Leptin exhibited strong positive correlations with BMI (*r* = 0.695, *p* < 0.001), WHR (*r* = 0.422, *p* = 0.001), WHtR (*r* = 0.628, *p* < 0.001, Figure 1), HOMA-IR (*r* = 0.408, *p* = 0.001, Figure 1), triglycerides (*r* = 0.273, *p* < 0.05), non-HDL-C (*r* = 0.274, *p* < 0.05, Figure 1), and BDNF levels (*r* = 0.471, *p* < 0.01, Figure 1), and a negative correlation with adiponectin (*r* = −0.330, *p* < 0.01).

**Figure 2:**
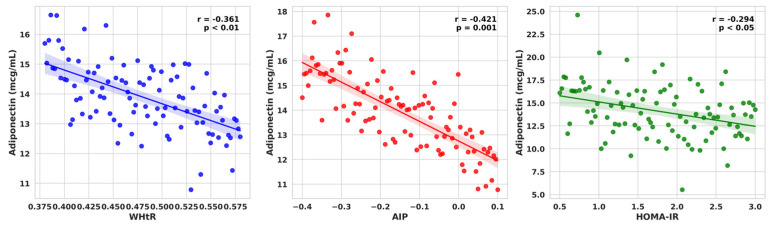
Associations of Adiponectin with WHtR, AIP and HOMA-IR in the Whole Study Group.

**Figure 3:**
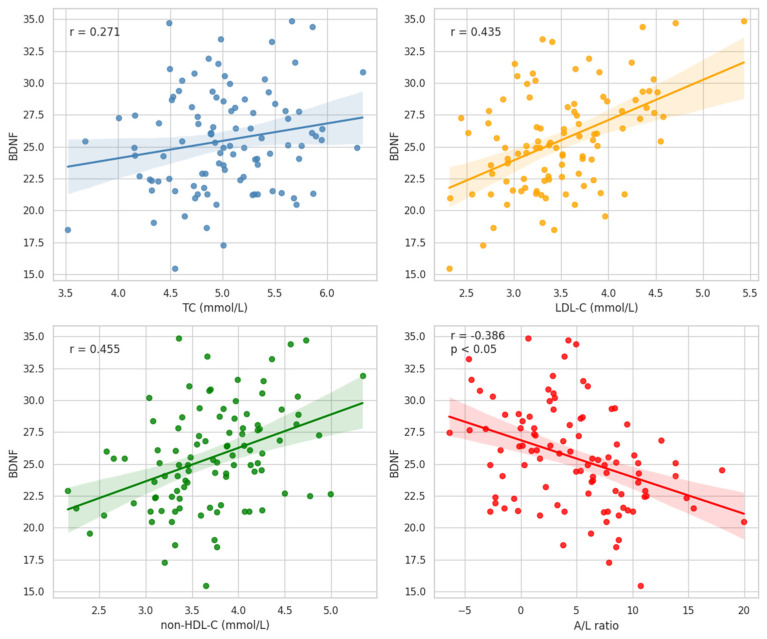
Associations of BDNF with TC, LDL-C, non-HDL-C and A/L ratio in the Whole Study Group.

Adiponectin showed inverse associations with anthropometric indices—BMI (*r* = −0.348, *p* < 0.01), WHR (*r* = −0.300, *p* < 0.05) and WHtR (*r* = −0.361, *p* < 0.01, Figure 2); with triglycerides (*r* = −0.411, *p* < 0.01), AIP (*r* = −0.421, *p* = 0.001, Figure 2), and HOMA-IR (*r* = −0.294, *p* < 0.05, Figure 3).

BDNF demonstrated positive correlations with TC (*r* = 0.271, *p* < 0.05), LDL-C (*r* = 0.435, *p* < 0.01), non-HDL-C (*r* = 0.455, *p* < 0.01), and negative relationship with A/L ratio (*r* = −0.386, *p* < 0.05) (Figure 3), suggesting an association with adiposity-related lipid alterations.

**Figure 4:**
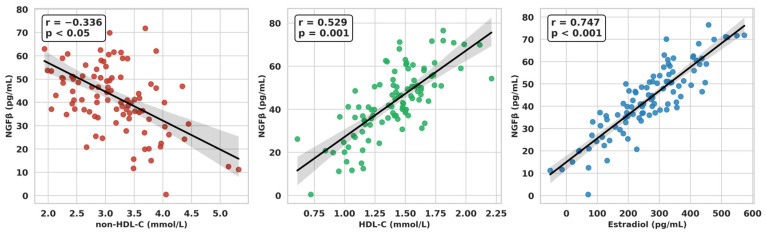
Associations of NGFβ with non-HDL-C, HDL-C and Estradiol in the Whole Study Group.

In contrast, NGFβ correlated negatively with non-HDL-C (*r* = −0.336, *p* < 0.05) and positively with HDL-C (*r* = 0.529, *p* = 0.001), (Figure 4). Importantly, NGFβ also showed a significant positive correlation with serum estradiol levels (*r* = 0.747, *p* < 0.001, Figure 4), indicating a potential link between neurotrophin signaling and ovarian steroidogenesis in PCOS.

### 2.6. Multivariate Regression Analysis

Given the mathematical overlap between BMI and VAI, two separate regression models were performed to avoid collinearity.

In Model 1 (anthropometric model), BMI, WHtR, HOMA-IR, and lipid parameters were evaluated as predictors of circulating neurotrophins. In Model 2 (VAI-based model), VAI replaced BMI and WHtR as an integrated marker of visceral adiposity. In this model, VAI (β = 0.312, *p* = 0.004) and non-HDL-C (β = 0.285, *p* = 0.011) remained independently associated with BDNF, whereas HDL-C (β = 0.398, *p* = 0.002) and estradiol (β = 0.547, *p* < 0.001) were independently associated with NGFβ.

### 2.7. Correlation Network and Heatmap Visualization

**Figure 5:**
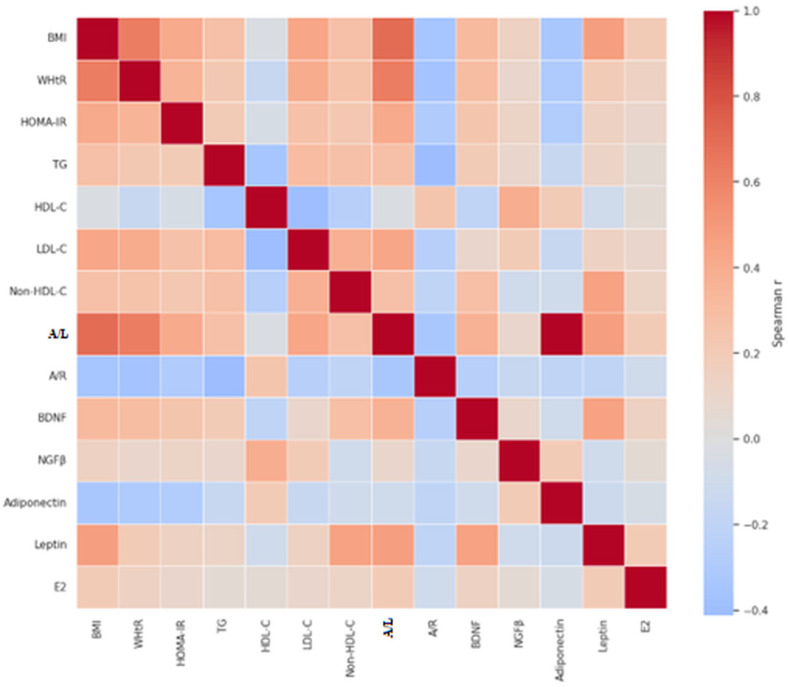
Correlation Heatmap of Neurotrophins, Adipokines, Anthropometric and Metabolic Markers. Colors represent the strength and direction of Spearman correlations (blue = negative, red = positive).

Spearman correlation analyses were performed to evaluate pairwise relationships among key anthropometric, metabolic, adipokine, and neurotrophin markers, including BMI, WHtR, HOMA-IR, lipid fractions (TG, HDL-C, LDL-C, non-HDL-C), BDNF, NGFβ, adiponectin/leptin (A/L) and adiponectin/resistin (A/R) ratios, and estradiol. A heatmap (Figure 5) was generated to visualize the strength and direction of all correlations, highlighting clusters of positive and negative associations. The correlation network illustrates integrated relationships, showing that high VAI clusters with BDNF and atherogenic lipids, whereas NGFβ clusters with HDL-C and estradiol, suggesting divergent neurotrophin–adipokine patterns according to cardiometabolic risk in PCOS. These visualizations complement the regression analyses, providing a holistic view of interrelated metabolic, hormonal, and neurotrophin alterations.

## 3. Discussion

In the present study, women with higher VAI exhibited markedly adverse anthropometric and metabolic profiles, including increased BMI, WHR, and WHtR, reflecting pronounced central obesity, together with elevated FIRI and HOMA-IR, indicative of enhanced IR. These findings are consistent with substantial evidence demonstrating that visceral adiposity, rather than generalized obesity, is a principal driver of cardiometabolic risk in PCOS. Previous studies have shown that WHtR and other indices of central fat distribution outperform BMI in predicting IR, dyslipidemia, and cardiovascular risk in women with PCOS [26,27]. Consistent with this, reviews highlight visceral fat as a major driver of dyslipidemia and cardiometabolic risk in PCOS, largely independent of total adiposity [28,29].

The lipid abnormalities observed in the high-VAI group—characterized by elevated triglycerides, non-HDL cholesterol, and atherogenic indices alongside reduced HDL-C—reflect a classical atherogenic metabolic phenotype. These findings align with the original VAI validation studies, which demonstrated robust links between elevated VAI, insulin resistance, and atherogenic dyslipidemia [8,30]. Subsequent investigations in PCOS populations have confirmed that higher VAI values are closely related to unfavorable lipid patterns and increased cardiovascular risk markers [31,32], reinforcing the clinical utility of VAI as a surrogate marker of visceral adipose tissue dysfunction in this syndrome.

With regard to adipokines, leptin concentrations tended to be higher, while adiponectin levels were significantly lower in women with elevated VAI, resulting in substantially decreased adiponectin/leptin (A/L) and adiponectin/resistin (A/R) ratios. This adipokine imbalance is widely recognized as a hallmark of adipose tissue dysfunction in PCOS. A recent meta-analysis by Lin et al. focusing on nonobese women with PCOS demonstrated that leptin levels are significantly elevated, whereas adiponectin concentrations are consistently reduced compared to nonobese controls, indicating that these alterations occur independently of BMI and are associated with IR and metabolic risk [33]. Moreover, several studies suggest that the A/L ratio is a more sensitive marker of metabolic impairment than individual adipokine levels, particularly in PCOS cohorts [34,35]. The strong positive correlations observed in our study between leptin, anthropometric indices, IR, and atherogenic lipid parameters further support the central role of leptin as a mediator linking excess visceral adiposity to systemic metabolic dysregulation. Although these associations are expected based on known physiology, they serve as internal validation of cohort metabolic characterization and strengthen the interpretation of neurotrophin-related findings.

Our neurotrophin profiling revealed distinct patterns according to VAI-defined cardiometabolic risk. Circulating BDNF concentrations were higher in women with elevated VAI and showed positive associations with leptin and atherogenic lipid fractions. Although data on BDNF in PCOS are heterogeneous, accumulating evidence indicates that BDNF participates in metabolic regulation beyond its classical neurotrophic functions. Experimental studies have demonstrated that BDNF influences energy homeostasis, lipid metabolism, and adipokine expression in adipose tissue [36,37,38,39]. In human studies, some reports describe reduced BDNF levels in obese women with PCOS [39,40], whereas others have observed elevated circulating BDNF in conditions characterized by metabolic stress, dyslipidemia, or IR [41,42]. Our findings support the latter perspective and suggest that increased BDNF in high-VAI women may represent a compensatory response to visceral adiposity-related metabolic stress, potentially modulated by dyslipidemia and leptin signaling.

In contrast to BDNF, NGFβ levels were higher in women with low VAI and were favorably associated with HDL-C, non-HDL-C, and estradiol concentrations, suggesting a potentially protective neurotrophic–endocrine profile in lower-risk PCOS phenotypes. NGF is well established as a key regulator of ovarian physiology, follicular development, and steroidogenesis [43]. Experimental models have shown that alterations in ovarian NGF expression affect follicular morphology, ovulation, and steroid hormone production [44,45]. Human data further support a role for NGF in ovarian endocrine regulation, including modulation of estrogen synthesis [46]. The strong positive correlation between NGFβ and estradiol observed in our cohort is therefore consistent with the concept that neurotrophin signaling interfaces with ovarian steroidogenic pathways and may contribute to more favorable endocrine and metabolic profiles in PCOS.

The use of separate regression models strengthens the robustness of the findings by minimizing collinearity between composite (VAI) and individual anthropometric variable.

Taken together, our results demonstrate that VAI-defined cardiometabolic risk in PCOS is accompanied by distinct and opposing neurotrophin and adipokine signatures. Elevated BDNF and decreased A/L ratio in high-VAI individuals reflect adiposity-related metabolic stress and dyslipidemia, whereas higher NGFβ levels in low-VAI women may indicate protective neurotrophic and endocrine modulation. The correlation heatmap and network further highlight these interconnections, showing that BDNF clusters with VAI and atherogenic lipid fractions, consistent with previous reports linking circulating BDNF to obesity-related dyslipidemia in individuals with IR [47,48]. Conversely, NGFβ clustered with estradiol and favorable lipid parameters, supporting a potential protective role in ovarian endocrine regulation and metabolic homeostasis. This observation is consistent with human and translational evidence demonstrating that NGF signaling interfaces with ovarian steroidogenesis and follicular function, including estrogen synthesis, and is implicated in the pathophysiology of PCOS [44,46]. The A/L ratio emerged as a key integrative marker linking insulin resistance, dyslipidemia, and visceral adiposity, consistent with evidence that adipokine ratios better capture adipose dysfunction than individual adipokine levels [49,50]. These findings highlight the interplay between visceral fat, adipokine imbalance, neurotrophin signaling, and cardiometabolic risk in PCOS, supporting a compensatory neurotrophin–adipokine network in response to metabolic and endocrine dysregulation.

A mechanistic framework may explain the divergent neurotrophin profiles across VAI-defined phenotypes. In high visceral adiposity, adipocyte hypertrophy and lipid overflow drive insulin resistance, dyslipidemia, and altered adipokine secretion, notably reduced adiponectin and relatively elevated leptin. Leptin can interact with central and peripheral BDNF pathways, potentially upregulating BDNF as a compensatory response to metabolic stress [51]. Central BDNF exerts anorexigenic effects and may promote weight regulation, whereas peripheral BDNF dynamics could reflect metabolic adaptation rather than energy restriction per se [52]. Epigenetic mechanisms, including DNA methylation, may further modulate BDNF and other metabolic genes in response to chronic metabolic stress [53]. Chronic low-grade inflammation, mediated by TNF-α and IL-6, likely contributes to adipokine and neurotrophin dysregulation, while nutritional factors such as DHA and other omega-3 fatty acids may enhance BDNF expression and attenuate inflammatory signaling [54]. Collectively, these layers support a complex integrative model in which metabolic, inflammatory, nutritional, and epigenetic inputs converge to shape neurotrophin–adipokine interactions. The neurotrophin–adipokine interplay in PCOS likely reflects a multifactorial network integrating metabolic, inflammatory, nutritional, and epigenetic signals [55].

Conversely, NGFβ aligns with favorable lipid profiles and estradiol levels, consistent with its role in follicular development and ovarian steroidogenesis. In lower-risk PCOS phenotypes, preserved NGFβ–estradiol interactions may support endocrine and metabolic stability, whereas disruption of this axis in high-VAI states could link visceral adiposity to hormonal imbalance.

**Figure 6:**
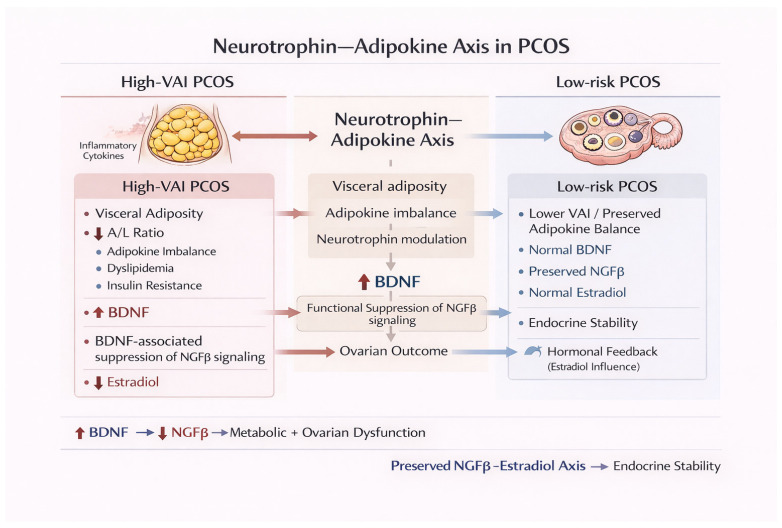
A hypothesis-generating conceptual model derived from the present findings and intended for experimental validation.

Based on these findings, we propose a “neurotrophin–adipokine axis” in PCOS: visceral adiposity induces an adipokine imbalance (reduced A/L ratio), which modulates circulating neurotrophins. Elevated BDNF reflects metabolic stress-related signaling associated with dyslipidemia, while preserved NGFβ supports ovarian steroidogenesis and favorable lipid handling (Figure 6). Future studies using adipose and ovarian cell models, along with longitudinal clinical cohorts, are needed to test whether modulation of this axis can influence cardiometabolic and reproductive outcomes in PCOS.

As the study did not include non-PCOS controls, it remains unclear whether the observed correlations reflect adiposity-driven mechanisms alone or interactions specific to the PCOS phenotype.

Several limitations should be acknowledged. The cross-sectional design precludes causal inferences between VAI, adipokines, neurotrophins, and metabolic parameters, and longitudinal studies are needed to clarify temporal relationships. Another important limitation is the absence of age-matched non-PCOS control groups, with and without central obesity. Therefore, our findings cannot disentangle the independent contributions of PCOS per se from those related to visceral adiposity. The results should thus be interpreted as reflecting risk stratification within PCOS rather than differences between PCOS and healthy women. Circulating BDNF and NGFβ may not fully reflect tissue-specific expression or local signaling in adipose tissue, ovary, or the central nervous system. Single-plex ELISA assays were employed, and all measurements were performed in duplicate under standardized laboratory conditions. Nonetheless, the use of multiplex platforms in future studies may further reduce inter-assay variability and enhance analytical robustness. In addition, while VAI represents a validated surrogate marker of visceral adiposity, direct imaging-based quantification (e.g., MRI or CT) was not performed. Given that physical activity, dietary composition, and psychological stress are known modulators of circulating BDNF levels and adipokine profiles, future studies incorporating standardized lifestyle assessment tools would allow more precise adjustment for these potentially confounding variables. Furthermore, detailed cardiovascular phenotyping (e.g., echocardiography or vascular function assessment) was not performed and would strengthen the clinical translational relevance of future investigations. Although VAI has been validated in Caucasian populations and widely used in research, no universally accepted clinical cut-off values are endorsed by international guidelines. Thus, VAI should be considered a surrogate research marker, and the use of the 1.9 threshold may limit direct clinical generalizability of our findings. Finally, the cohort size and its clinical characteristics may limit generalizability to broader PCOS populations. Nevertheless, this integrated evaluation of neurotrophins and adipokines in relation to VAI provides novel insights into cardiometabolic risk signatures in PCOS.

## 4. Materials and Methods

### 4.1. Study Design and Participants

This cross-sectional study included 100 women diagnosed with polycystic ovary syndrome (PCOS) according to the Rotterdam criteria 2003 [56]. Participants were recruited from the Clinic of Endocrinology and Metabolic Diseases, “St. George” University Hospital of Plovdiv, Bulgaria between June 2020 and June 2023. Women were stratified into lower-risk (low-VAI, n = 50) and higher-risk (high-VAI, n = 50) groups based on the Visceral Adiposity Index (VAI). VAI was calculated according to the sex-specific formula proposed by Amato et al. The cut-off value of 1.9 for women was applied to define increased visceral adipose tissue dysfunction and cardiometabolic risk, as originally described and validated in a Caucasian population. Although this threshold has been widely used in epidemiological and clinical research, VAI cut-off values are not universally standardized across populations and are not currently incorporated into formal international clinical guidelines. Therefore, in the present study, VAI was used as a research-based surrogate marker for visceral adiposity dysfunction rather than as a diagnostic criterion [57].

Women aged 18–35 years with a confirmed diagnosis of PCOS who had not received hormonal or metabolic treatments in the preceding six months were eligible for inclusion. Participants were excluded if they were pregnant or lactating, had type 2 diabetes mellitus, cardiovascular disease, chronic inflammatory or autoimmune disorders, thyroid or adrenal pathology, or were using insulin-sensitizing or lipid-lowering agents (e.g., metformin, statins) or hormonal contraceptives, or if they were active smokers.

All participants provided written informed consent. The study protocol was approved by the Medical University of Plovdiv Institutional Ethics Committee (protocol code 2444/26 October 2020), in accordance with the Declaration of Helsinki.

### 4.2. Anthropometric and Clinical Assessment

Body weight and height were measured using standard calibrated scales and stadiometers (Seca GmbH & Co. KG, Hamburg, Germany). BMI was calculated as weight (kg) divided by height squared (m^2^). Waist and hip circumferences were measured with a flexible tape at the midpoint between the lowest rib and iliac crest (waist) and at the widest part of the hips, respectively (Seca GmbH & Co. KG, Hamburg, Germany). WHR and WHtR were calculated accordingly. Blood pressure was measured in the seated position after a 5 min rest using an automated sphygmomanometer (Microlife, Widnau, Switzerland).

### 4.3. Biochemical and Hormonal Measurements

Venous blood samples for laboratory analyses were collected under standardized conditions in the early morning, following an overnight fast of 12 h, during the follicular phase of the menstrual cycle (2nd to 5th day after a spontaneously occurring cycle). Samples for the determination of fasting blood glucose (FBG) and fasting insulin (FIRI), lipid profile, standard hormonal parameters, neurotrophins, and adipokines were processed at the Central Clinic Laboratory, St. George University Hospital, Plovdiv, Bulgaria. Serum was separated by centrifugation at 3000 rpm for 10 min and stored at −80 °C until analysis.

#### 4.3.1. Glucose Metabolism

Fasting serum glucose was measured using the glucose oxidase-peroxidase (GOD-POD) technique. Serum insulin levels were assessed with a chemiluminescent immunoassay (CLIA) kit (Beckman Coulter, Chaska, MN, USA). The assay exhibited reliable analytical characteristics, including dilution recovery between 96% and 104%, a sensitivity of 0.03 μIU/mL, intra-assay CV ranging from 2.0% to 4.2%, and inter-assay CV between 3.1% and 5.6%. The method showed no cross-reactivity with bilirubin (10 mg/dL), triglycerides (20.32 mmol/L), or C-peptide (20,000 pmol/L), with a reference interval of 1.9–23.0 μIU/mL.

Insulin resistance was evaluated using the homeostasis model assessment (HOMA-IR), calculated by multiplying fasting insulin (µU/mL) by fasting blood glucose (mmol/L) and dividing the result by 22.5.

#### 4.3.2. Lipid Profile

Serum lipid concentrations were determined enzymatically. Total cholesterol (TC) was determined using the ChOD-PAP method, while triglycerides (TG) were measured by the GPO-PAP technique. HDL cholesterol (HDL-C) was quantified after precipitation with MgSO_4_-dextran sulfate. All measurements were carried out with reagents from Schneiders Medizintechnik, Zwolle, Netherlands on a Delta Kone autoanalyzer (Kone Instruments, Espoo, Finland). Low-density lipoprotein cholesterol (LDL-C) was estimated using the Friedewald formula, while non-HDL cholesterol was obtained by subtracting HDL-C from total cholesterol (TC). The Atherogenic Index of Plasma (AIP) was calculated as the logarithm of the TG-to-HDL-C ratio (log[TG/HDL-C]).

#### 4.3.3. Neurotrophins and Adipokines

Serum adipokine and neurotrophin levels were quantified using commercially available ELISA kits, following the protocols provided by the manufacturers.

Leptin concentrations were assessed using a solid-phase human ELISA kit (DRG Instruments GmbH, Marburg, Germany), with a sensitivity of 0.2 ng/mL, intra-assay CV below 8.7%, and inter-assay CV under 5.4%. Adiponectin levels were quantified with a human ELISA kit (BioVendor – Laboratorní medicína a.s., Brno, Czech Republic), showing a sensitivity of 26 ng/mL, intra-assay CV less than 5.9%, and inter-assay CV below 7.0%. Resistin was measured using a competitive solid-phase human EIA kit (Phoenix Pharmaceuticals, Inc., Burlingame, CA, USA), with a sensitivity of 1.16 ng/mL, intra-assay CV under 14.0%, and inter-assay CV less than 5.0%.

Brain-derived neurotrophic factor (BDNF) concentrations were measured using a human BDNF ELISA kit (R&D Systems, Minneapolis, MN, USA), with a sensitivity of 0.01 ng/mL, intra-assay CV < 6.5%, and inter-assay CV < 7.2%. Nerve growth factor beta (NGFβ) levels were determined using a human NGFβ ELISA kit (Abcam, Cambridge, UK) with a sensitivity of 1 pg/mL, intra-assay CV < 6%, and inter-assay CV < 6.5%. All serum samples were stored at −80 °C until analysis. All samples were analyzed in duplicate within the same analytical run whenever possible, using kits from the same lot number. Samples underwent a single freeze–thaw cycle to minimize analytical variability.

Adipokine ratios (A/L, A/R) were calculated for each participant.

#### 4.3.4. Hormonal Parameters

Serum luteinizing hormone (LH) and follicle-stimulating hormone (FSH) levels were quantified using automated chemiluminescent immunoassays (CLIA) (Beckman Coulter, Inc., Brea, CA, USA) based on a sandwich immunochemical principle. The LH assay had a sensitivity of 0.2 IU/L, dilution recovery of 96.9–102.8%, intra-assay CVs of 3.6–3.8%, and inter-assay CVs of 4.3–6.4%, with no detectable cross-reactivity. The reference range for LH in the follicular phase was 2.12–10.89 IU/L. The FSH assay demonstrated a sensitivity of 0.2 mIU/mL, dilution recovery of 96.6–104.3%, intra-assay CVs of 3.1–4.3%, and inter-assay CVs of 4.3–5.6%, with a follicular-phase reference range of 3.85–8.78 mIU/mL. Serum estradiol (E2) concentrations were measured using a chemiluminescent immunoassay (CLIA) kit (Beckman Coulter, Inc.) based on a competitive immunochemical principle. The assay demonstrated dilution recovery ranging from 97% to 118% and an analytical sensitivity of 73 pmol/L. Intra-assay coefficients of variation (CVs) ranged from 3.1% to 19.7%, while inter-assay CVs ranged from 5.0% to 20.0%. The method showed high analytical specificity, with no detectable cross-reactivity with bilirubin, triglycerides, hemoglobin, estriol, aldosterone, or testosterone. The reference range for estradiol during the follicular phase of the menstrual cycle was 99–448 pmol/L. The LH/FSH ratio was calculated for all participants.

Serum androgen and sex hormone-binding globulin (SHBG) levels were quantified using automated chemiluminescent immunoassays (CLIA), following the manufacturers’ protocols. Total testosterone (TT) was measured with a CLIA kit (Beckman Coulter, Inc., Brea, CA, USA), featuring an analytical sensitivity of 0.35 ng/mL, dilution recovery of 96–115%, intra-assay CVs of 1.7–3.9%, and inter-assay CVs of 4.2–7.1%. The assay demonstrated high specificity, with no significant cross-reactivity, and a reference interval for women of 0.1–0.75 ng/mL.

Serum androstenedione (A4) was measured using a CLIA kit (catalog no. L2KAO2; Siemens Healthcare Diagnostics, Tarrytown, NY, USA), with a sensitivity of 1.0 nmol/L, intra-assay CVs ranging from 6.2% to 15.1%, and total imprecision between 8.5% and 17.8%. The reference range for women in the follicular phase was 0.75–3.1 ng/mL. Dehydroepiandrosterone sulfate (DHEA-S) levels were quantified using a CLIA kit (Beckman Coulter, Inc.), with a sensitivity < 2 μg/dL, dilution recovery of 94.8–116.2%, intra-assay CVs of 1.6–8.3%, and inter-assay CVs of 4.4–11.3%; age-specific reference ranges were applied.

SHBG levels were determined using the Access 2 Immunoassay System (Beckman Coulter, Inc.), with an analytical sensitivity of 0.33 nmol/L and a measurable range up to 180 nmol/L. Intra- and inter-assay CVs were typically below 10%. The free androgen index (FAI) was calculated as: TT (nmol/L) × 100/SHBG (nmol/L).

### 4.4. Statistical Analysis

Statistical analyses were conducted using SPSS software (version 21.0; IBM Corp., Armonk, NY, USA). The Kolmogorov–Smirnov test was applied to assess data distribution. Variables not following a normal distribution, such as LH concentrations and the LH/FSH ratio, were log10-transformed before analysis. Data with normal distribution are presented as mean ± standard deviation (SD), while non-normally distributed variables are reported as median (interquartile range). Group comparisons were performed using either Student’s *t*-test or the Mann–Whitney U test, depending on data distribution. All tests were two-tailed, and a *p*-value less than 0.05 was considered statistically significant.

To minimize potential multicollinearity resulting from the mathematical incorporation of BMI, waist circumference, triglycerides, and HDL-C into the VAI formula, composite and component variables were not entered simultaneously into the same regression model. Instead, two separate multivariate linear regression models were constructed. The first model (basic anthropometric model) included BMI, WHtR, HOMA-IR, and lipid fractions as independent predictors. The second model (VAI-based model) incorporated VAI as a composite marker of visceral adiposity, together with non-HDL-C, HOMA-IR, and estradiol. The models were analyzed independently to reduce collinearity bias, and multicollinearity was assessed using variance inflation factors (VIFs), with only models demonstrating acceptable VIF values retained for final interpretation.

Correlation analyses were conducted to explore associations between anthropometric indices, metabolic parameters, adipokines, and neurotrophins. Spearman’s rank correlation coefficients were used for correlation network and heatmap visualization to account for non-normal distributions and to capture monotonic relationships among variables. Pearson correlation coefficients were used for selected pairwise associations displayed as scatter plots.

To provide an integrative overview of interrelationships among variables, correlation heatmap and network analyses were generated based on Spearman correlation matrices. The heatmap visualizes the strength and direction of pairwise correlations among key markers, including BMI, WHtR, VAI, HOMA-IR, lipid fractions, adipokine ratios (A/L and A/R), BDNF, NGFβ, and estradiol. The correlation network highlights clusters of closely related variables and opposing patterns according to cardiometabolic risk profiles.

## 5. Conclusions

Our study suggests that visceral adiposity-driven cardiometabolic risk in women with PCOS is associated with distinct neurotrophin and adipokine signatures. High VAI is linked to elevated BDNF levels and unfavorable adiponectin/leptin and adiponectin/resistin ratios, reflecting adiposity-related metabolic stress and insulin resistance. Conversely, higher NGFβ concentrations in low-VAI women may indicate protective neurotrophic and endocrine modulation, including favorable lipid handling and estradiol regulation. These findings highlight a dual neurotrophin–adipokine axis as a potential integrative mediator of metabolic and endocrine alterations in PCOS. Importantly, this study moves beyond confirming the established association between visceral obesity and metabolic abnormalities in PCOS by identifying distinct neurotrophin signatures linked to VAI-defined cardiometabolic risk. By integrating neurotrophins with adipokine ratios and lipid indices within a single well-characterized PCOS cohort, the present work provides a multidimensional risk stratification framework that may refine future biomarker-driven approaches in PCOS management.

Future longitudinal and mechanistic studies are warranted to explore whether modulation of neurotrophin and adipokine profiles could serve as therapeutic targets to mitigate cardiometabolic and reproductive risks in this population.

## Figures and Tables

**Table 1:** Age, anthropometric and clinical parameters in the two studied groups of women with PCOS.

Parameter	Low-VAI PCOS (n = 50)	High-VAI PCOS (n = 50)
Age (years)	23.87 ± 4.05	24.36 ± 5.24 NS
Height (cm)	166.48 ± 8.33	166.64 ± 5.85 NS
Weight (kg)	61.75 ± 12.19	76.84 ± 16.12 ***
BMI (kg/m^2^) ∆	22.21 ± 3.81	27.64 ± 5.57 ***
Waist (cm) ∆	71.17 ± 7.83	88.85 ± 14.27 ***
Hip (cm)	97.30 ± 9.51	105.15 ± 11.07 **
WHR	0.73 ± 0.05	0.84 ± 0.08 ***
WHtR	0.43 ± 0.04	0.53 ± 0.09 ***
SBP (mmHg)	109.57 ± 12.52	116.09 ± 11.05 *
DBP (mmHg)	71.96 ± 9.14	73.04 ± 9.16 NS

NS—not significant (*p* > 0.05); *—*p* < 0.05; **—*p* < 0.01; ***—*p* < 0.001; ∆—these parameters are components of VAI calculation.

**Table 2:** Adipokines and neurotrophins levels; values of adiponectin-to-leptin ratio (A/L) and adiponectin-to-resistin ratio (A/R) in the two studied groups of women with PCOS.

Parameter	Low-VAI PCOS (n = 50)	High-VAI PCOS (n = 50)
Leptin (ng/mL)	27.43 ± 13.27	34.06 ± 19.92 NS
Adiponectin (mcg/mL)	17.52 ± 8.70	9.95 ± 4.71 ***
Resistin (ng/mL)	5.47 ± 2.42	6.85 ± 4.62 NS
BDNF (ng/mL)	23.03 ± 3.05	26.11 ± 4.14 *
NGFβ (pg/mL)	51.40 ± 29.20	39.15 ± 4.38 *
A/L	1.19 ± 0.84	0.59 ± 0.46 **
A/R	26.80 ± 14.66	14.61 ± 8.21 ***

NS—not significant (*p* > 0.05); *—*p* < 0.05; **—*p* < 0.01; ***—*p* < 0.001.

**Table 3:** Glucose homeostasis and insulin resistance parameters; lipid profile and atherogenic indices in the two studied groups of women with PCOS.

Parameter	Low-VAI PCOS (n = 50)	High-VAI PCOS (n = 50)
FBG (mmol/L)	4.71 ± 0.47	4.90 ± 0.66 NS
FIRI (μIU/mL)	5.97 ± 2.26	9.48 ± 4.44 **
HOMA-IR	1.27 ± 0.59	2.13 ± 1.17 **
TC (mmol/L)	4.48 ± 1.14	4.47 ± 0.75 NS
LDL-C (mmol/L)	2.57 ± 1.11	2.77 ± 0.69 NS
HDL-C (mmol/L) ∆	1.67 ± 0.49	1.15 ± 0.25 ***
TG (mmol/L) ∆	0.54 ± 0.16	1.18 ± 0.44 ***
Non-HDL-C	2.82 ± 1.14	3.31 ± 0.73 *
AIP	−0.19 ± 0.12	0.03 ± 0.15 ***

NS—not significant (*p* > 0.05); *—*p* < 0.05; **—*p* < 0.01; ***—*p* < 0.001; AIP—Atherogenic Index of Plasma; ∆—these parameters are components of VAI calculation.

**Table 4:** Hormonal parameters in the two studied groups of women with PCOS.

Parameter	Low-VAI PCOS (n = 50)	High-VAI PCOS (n = 50)
Log LH (IU/L)	0.80 ± 0.26	0.79 ± 0.36 NS
FSH (mIU/mL)	5.90 ± 1.26	5.46 ± 2.12 NS
Log LH/FSH	0.04 ± 0.26	0.09 ± 0.34 NS
E2 (pg/mL)	314.67 ± 176.14	228.68 ± 183.03 NS
Total testosterone (ng/mL)	0.66 ± 0.17	0.70 ± 0.20 NS
Androstenedione (ng/mL)	4.16 ± 2.34	3.75 ± 1.44 NS
DHEA-S (μg/dL)	275.77 ± 122.91	289.72 ± 103.67 NS
SHBG (nmol/L)	46.78 ± 10.25	37.20 ± 18.64 NS
FAI	5.01 ± 2.47	9.43 ± 5.46 NS

NS—not significant (*p* > 0.05); FAI—free androgen index.

## Data Availability

The data that support the findings of this study are available from the corresponding author upon reasonable request. Due to ethical restrictions and participant confidentiality, the dataset is not publicly available.

## Abbreviations

The following abbreviations are used in this manuscript:

PCOS	Polycystic Ovary Syndrome
IR	Insulin Resistance
BMI	Body Mass Index
HOMA-IR	Homeostatic Model Assessment for Insulin Resistance
WHtR	Waist-to-Height Ratio
AIP	Atherogenic Index of Plasma
HDL-C	High-Density Lipoprotein Cholesterol
LDL-C	Low-Density Lipoprotein Cholesterol
TG	Triglycerides
A/L	Adiponectin-to-Leptin Ratio
A/R	Adiponectin-to-Resistin Ratio
VAI	Visceral Adiposity Index
BDNF	Brain-derived Neurotrophic Factor
NGFβ	Nerve Growth Factor-β
ELISA	Enzyme-Linked Immunosorbent Assay
LH	Luteinizing hormone
FSH	Follicle-Stimulating Hormone
E2	Estradiol
TT	Total Testosterone
SHBG	Sex Hormone-Binding Globulin
DHEA-S	Dehydroepiandrosterone Sulfate
FAI	Free Androgen Index
